# DNA methylation landscapes in diploid and allotetraploid species in peanut

**DOI:** 10.1093/plphys/kiaf175

**Published:** 2025-05-03

**Authors:** Nilesh D Gawande

**Affiliations:** Assistant Features Editor, Plant Physiology, American Society of Plant Biologists; Department of Biological Sciences and Engineering, Indian Institute of Technology Gandhinagar, Palaj, Gujarat 382355, India

Polyploidization is an evolutionary mechanism that contributes to plant adaptation, diversification, and speciation (Wendel et al. 2016). There are two types of polyploidy: autopolyploidy, where similar genomes duplicate, and allopolyploidy, which results from the combination of two or more genomes of ancestral species (Heslop-Harrison et al. 2023). Examples of allopolyploids include allohexaploids like wheat (*Triticum aestivum*), and allotetraploids, such as *Brassica napus* and cotton (*Gossypium hirsutum*). Similar genes from the genomes of allopolyploid species are called homeologs, which often have varying levels of gene expression, possibly due to the interaction between their subgenomes and epigenetic changes incorporated during their formation.

DNA methylation is a major epigenetic change that contributes to gene expression regulation. CG is the common DNA methylation site in animals, whereas in plants, DNA methylation occurs at three different sites: CG, CHG, and CHH, where H can be A, T, or C (Zhang et al. 2018). Various enzymes such as METHYLTRANSFERASE1 (MET1), CHROMOMETHYLASE3 (CMT3), DOMAINS REARRANGED METHYLTRANSFERASE2 (DRM2), and CHROMOMETHYLASE2 (CMT2) maintain DNA methylation at these sites. In addition, RNA-directed DNA methylation (RdDM) is another pathway for DNA methylation (He et al. 2011). In allopolyploid species such as bread wheat and cotton, DNA methylation and histone modifications influence the separation of subgenomes, leading to variability in the gene expression levels of homeologs as well as transposable elements.

Peanut (*Arachis hypogea* L.) is an important oil seed crop and an allotetraploid formed by the combination of two diploid ancestral genomes: *Arachis duranensis* and *Arachis ipaensis* (Chen et al. 2019). Several studies have focused on varied gene expression levels among the homeologs in peanuts; however, the role of epigenetic modifications such as DNA methylation in these expression biases is not well studied. Therefore, investigation of the DNA methylation in the diploid ancestors and allotetraploid peanut species will give insight into the effect of these modifications on genome evolution and domestication.

In this issue of *Plant Physiology*, Yu et al. (2025) analyzed the DNA methylation in the diploid peanut ancestral species *A. duranensis* (AA) and *A. ipaensis* (BB), and the tetraploid species *A. hypogea* (AABB). They found that at the subgenome level, the tetraploid peanut A subgenome exhibited higher CG and CHG methylation levels compared to the diploid ancestor A subgenome. Overall, in gene and flanking regions, tetraploid peanuts had increased CG and decreased CHH methylation levels compared to their diploid counterparts. However, in transposable elements, tetraploid peanut displayed higher CG and CHG methylation. This demonstrates the variation in DNA methylation landscapes in diploid and tetraploid species in different regions. The schematic representation for the study is provided in Figure 1.

**Figure 1.**
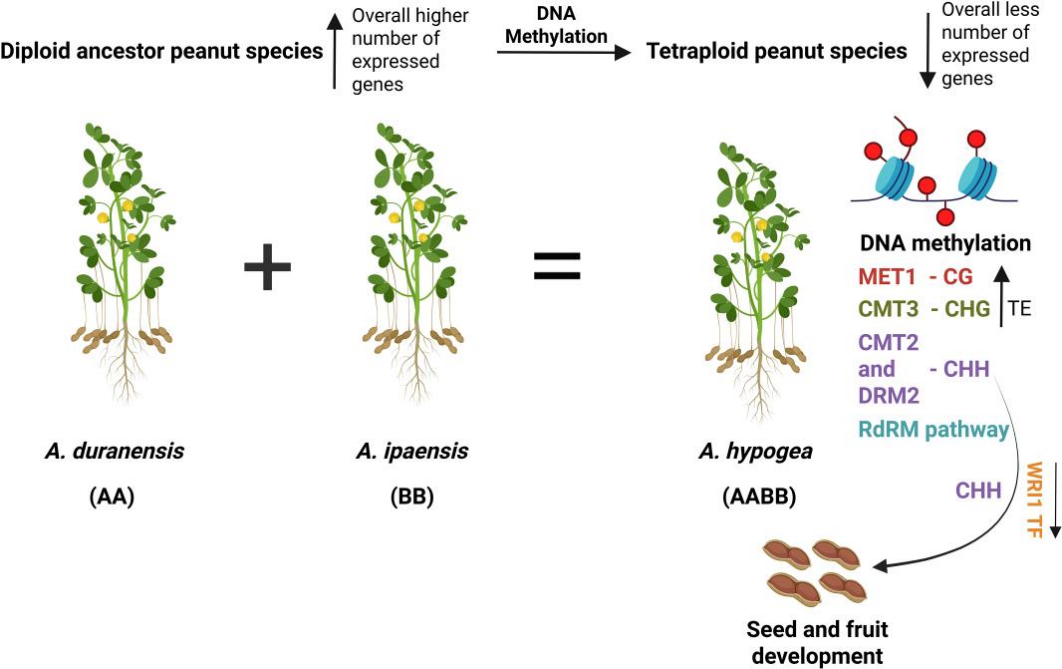
DNA methylation landscape in diploid and tetraploid species of peanut. Domesticated peanuts (*Arachis hypogaea*) are an allotetraploid (AABB) dervided from two diploid parents: *Arachis duranensis* (AA) and *Arachis ipaensis* (BB). A comparison of DNA methylation levels shows that diploid peanuts have overall higher number of expressed genes compared to tetraploid correlating with increased DNA methylation in tetraploid species. The authors showed that in the tetraploid, the A subgenome transposable elements have higher CG and CHG methylation than in the diploid parent. In the tetraploid, CHH methylation downregulates transcription factor WRINKLED1 (WRI1) and regulate the genes involved in seed and fruit development.

The authors investigated DNA methylation regulation by analyzing expression levels of methyltransferases, demethylases, and RdDM pathway genes using *Arabidopsis* homologs. Expression levels of the methyltransferases *MET1* responsible for CG, *CMT3* for CHG, and *CMT2* for CHH methylation had no significant changes in diploid and tetraploid peanuts. In contrast, the demethylase *ROS1b* exhibited higher expression in diploid peanuts, suggesting that the increased CG and CHG methylation in tetraploids is due to reduced demethylase expression. CHH methylation is originated from de novo methylation through the RdDM pathway. The key genes, such as *AGO4* and *DRM2*, involved in the RdDM pathway displayed reduced expression in tetraploid peanuts, indicating that the methylation changes in the tetraploid genome were also mediated through RdDM pathway. This indicates the involvement of CHH methylation through the RdDM pathway in peanut genome evolution. Furthermore, the differentially methylated regions (DMRs) between tetraploid and diploid (Ad) peanuts showed that tetraploid subgenomes had different methylation than ancestral diploid genomes.

The authors further analyzed the transcriptome of the diploid and tetraploid cultivars for homeologous gene expression. Diploid *A. duranensis* and *A. ipaensis* showed a higher number of expressed genes than tetraploid *A. hypogea*, demonstrating that the repression of genes in tetraploid species is due to the genetic changes happened as a result of polyploidization. Genome-wide expression analysis for homologous genes in tetraploid and its diploid ancestors demonstrated that most genes downregulated in the polyploid may reflect the need to balance increased gene copy number to maintain essential traits. A high percentage of differentially expressed genes were related to differentially methylated genes, suggesting changes in DNA methylation play a significant role in the expression of these genes in the polyploid.

The expression level dominance (ELD) compares the overall expression of homeologous gene pairs in hybrid polyploids to the expression levels of the corresponding genes in their parent species. In tetraploid peanuts, most of the homeologous gene pairs showed equivalent expression to their ancestor, while a significant portion had ELD, primarily influenced by changes in gene expression during the merging of subgenomes. CHG methylation levels negatively correlated with gene expression, indicating its role in shaping dominant expression patterns. Gene Ontology (GO) analysis revealed that ELD homeologous gene pairs were enriched in the biological functions related to organonitrogen compounds, seed germination regulation, photosynthesis, flavonoid biosynthesis, and fatty acid biosynthesis.

DNA methylation is crucial in plant developmental processes, including seed formation (Matilla 2020). Whole-genome bisulfite sequencing and RNA-seq analysis during seed development stages of tetraploid peanut showed that the tetraploid B subgenome had higher CG, CHG, and CHH methylation levels than the A subgenome. A significant increase in CHH methylation was observed during seed development, while CG and CHG methylation levels remained stable, which can be correlated with decreased demethylase expression. A higher number of differentially expressed genes were observed during early seed development, with fewer changes later, suggesting stability at later stages. Interestingly, CHH methylation has an important role in downregulating gene expression levels of the important transcription factor WRINKLED1 (WRI1), which is linked to seed and fruit development, suggesting a regulatory role of CHH methylation in peanut seed development.

In summary, this study provides an insight into the complex interactions between DNA methylation, gene expression, and the evolutionary trajectory of polyploid species to understand how allotetraploid peanuts diverged from their diploid progenitors. The observed differences in methylation can also help explain how gene expression dynamics are regulated post-polyploidization, affecting important traits such as seed development for agriculture and crop improvement. This study can be further extended to study the role of DNA methylation in enhancing the important agronomic traits in peanut as well as polyploid species. Moreover, other studies, such as investigating the role of long non-coding RNA (lncRNA) in DNA methylation, could be important future work for researchers working in peanuts.

## Data Availability

Not applicable.
